# Predictive Models for Kidney Offer Acceptance: Challenges and Strategies

**DOI:** 10.1155/joot/8243450

**Published:** 2026-01-09

**Authors:** Carlos Martinez, Md Nasir, Meghana Kshirsagar, Cass McCharen, Rae Shean, Juan Lavista Ferres, Rahul Dodhia, William B. Weeks

**Affiliations:** ^1^ Research Science, United Network for Organ Sharing, Richmond, Virginia, USA; ^2^ AI for Good Lab, Microsoft, Redmond, Washington, USA, microsoft.com

## Abstract

**Background:**

Predicting whether an organ offer will be accepted for transplantation remains challenging for several reasons, including large offer volumes, highly imbalanced observations (more declines than acceptances), and lack of information about the human decision‐making process. Offer acceptance models are used for risk‐adjusted program evaluations and policy development, but there is a lack of literature on baselines and best practices for predictive applications. We compared a suite of machine learning models, feature sets, and sampling procedures to identify performance impacts when training offer acceptance prediction models.

**Methods:**

We evaluated several kidney offer acceptance models from logistic regression to gradient boosted trees that were trained on donor and candidate characteristics. We then selected the best‐performing model and augmented training data with additional features (e.g., distance from the closest airport to the transplant hospital) or additional sampling procedures (e.g., undersampling).

**Results:**

Compared to the baseline logistic regression model (average precision: 0.0645), the XGBoost model offered the best performance improvement over the baseline (average precision: 0.0907). Including transportation‐related features in the model further improved model performance (average precision: 0.0940); however, we did not observe substantial model performance differences based on the sampling procedure used.

**Conclusions:**

Leveraging advanced machine learning models and incorporating nonclinical datapoints (like transportation distances) can improve transplant organ offer acceptance prediction models. However, we observed steep tradeoffs between precision and recall as captured in the low average precision scores despite deceptively high AUROCs (baseline AUROC 0.832). Our findings suggest that even the best‐performing models would not provide clear, equitable benefits over existing allocation policies. More research is needed before these models are practical for clinical implementation.

## 1. Introduction

Reviews of the transplant system operated by the Organ Procurement and Transplantation Network (OPTN) have called for increased modernization and technology integration [1]. Decision support tools powered by machine learning are being implemented both in healthcare and in transplant specifically [2, 3]. In the transplant setting, where complex decisions must be made quickly, these tools are intended to help decision makers reach their final verdicts efficiently.

**Figure 1:**
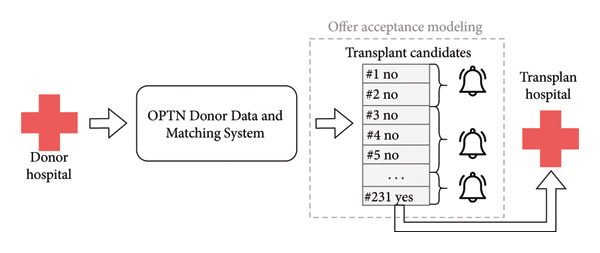
Simplified overview of the organ offer process for deceased donors. First, at a donor hospital, it will be decided to move forward with organ donation. An organ procurement organization (OPO) will collect information about the donor and enter it into the Organ Procurement and Transplantation (OPTN) Donor Data and Matching System (also known as DonorNet). Then it generates an ordered list of transplant candidates (known as a match run) based on established allocation policy that factors in donor and candidate characteristics. The OPO then sends out notifications to transplant hospitals for the candidates that an organ is available, and the transplant hospital must ultimately respond with a refusal (“No”) or acceptance (“Yes”). The OPO will notify batches of candidates at a time until the final acceptance is reached or no candidates accept. The organ will then be transported to the accepting transplant hospital. Our offer modeling in this work attempts to predict the probability of entering a “Yes” for each candidate.

The organ allocation process is depicted in Figure 1. Among the most pertinent questions during allocation is “Will this transplant hospital accept this organ for their patient?” This is especially relevant when the organ is of uncertain quality or has complicating circumstances, such as a liver donated after cardiac death (DCD). There have been attempts to improve allocation by establishing rules for when an organ may undergo expedited placement as well as criteria for establishing if a particular program is willing to accept difficult‐to‐place organs, but the results were not conclusive [4].

Previous studies in offer acceptance modeling emphasize the utility of big data processing tools [5] and modeling the individualized decision‐making process [6], but most are not aimed at the practical application of predictive models. For example, one study applies sampling procedures not just to the training dataset but also to the test dataset on which performance metrics were evaluated [5]; conclusions based on such an artificially inflated test set cannot be generalized to real‐world applications. Although used for retrospective inference instead of predictions, the Scientific Registry of Transplant Recipients (SRTR) routinely fits statistical models to historic offer data to evaluate whether a transplant program is accepting and transplanting offers at higher or lower rates than the national average while risk‐adjusting for the offers received [7]. Further, predictions for overall kidney nonuse are well‐studied and used within allocation policies [8, 9]. However, models trained for unseen offer acceptance predictions are less commonly developed.

In this work, we tested a suite of machine learning models trained on kidney offers to predict if the offer would be accepted or not. The kidney nonuse rate has steadily increased in recent years [10], and more equitable but geographically complex allocation policies have increased the resource burden for transplant programs [11]. As we developed our models, we emphasized the practical evaluation of their predictive performance with an expectation that the models would be used in decision support tools to expedite placements, decrease ischemic time, and result in more kidneys being successfully transplanted.

## 2. Materials and Methods

### 2.1. Data Collection

We collected data, including donor and candidate information at the time of organ offer, from all 217,950,819 deceased donor kidney offers sent between January 1, 2015, and June 23, 2022. All data were de‐identified by the OPTN before being uploaded to a secure cloud storage instance. If there was at least one acceptance on a particular match run (see Figure 1), then all offers up to and including the final acceptor were included in the final dataset; otherwise, only offers before the match was closed were included. All bypassed offers were removed, leaving 14,206,630 (6.5% of the total) for analysis. The donor and candidate characteristics used for prediction reflect standard variables used in transplant analyses, including those used for risk‐adjusting offer acceptance metrics by the SRTR [7], such as donor age and serum creatinine measurements. We did not include the sequence number of the candidate, as we anticipate predictions being used to reorder or bypass candidates, so we want to predict who would accept regardless of where they currently rank. A complete list of variables is included in Supporting Table S1.

### 2.2. Modeling and Experimental Setup

**Figure 2:**
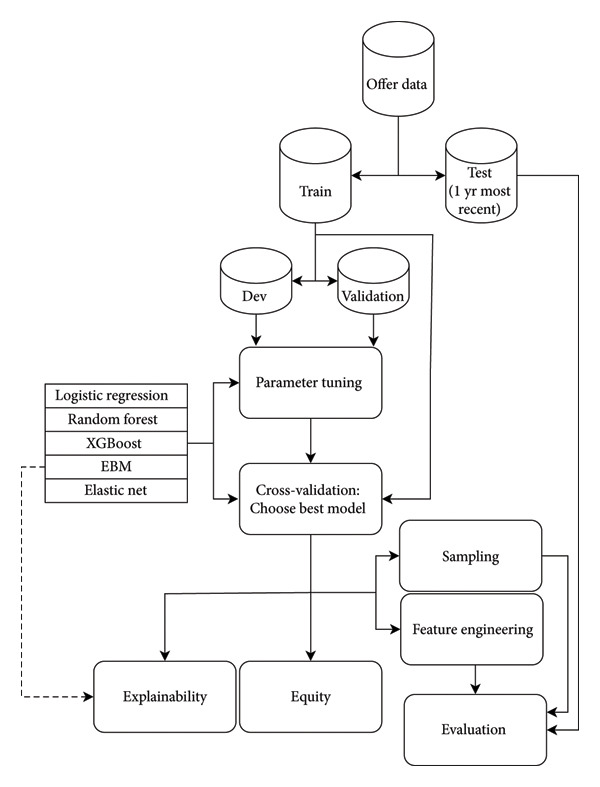
Model training and experimentation workflow. Offer data were partitioned into train and test sets. The train set was further split into development and validation sets for hyperparameter tuning for each model; the entire train set was then used with 10‐fold cross‐validation to select the optimal model architecture. The best model architecture was selected for further analysis of explainability, equity analyses of sensitive populations, and additional feature engineering before final performance evaluation with the hold‐out test set.

Model training and experimentation is outlined in Figure 2. The set of offers was split into training and test sets. 11,260,804 (80%) of the included offers were designated as the training set; the rest were reserved as the test set for evaluating final predictions. Since we want the test data to reflect the characteristics of data closest to when the model would be used, the test set consisted of 1 year of recent offer data from June 24, 2021, through June 23, 2022. Further, the training set was split into 10 approximately equal‐sized partitions for 10‐fold cross‐validation. Folds were partitioned at the donor level such that offers from a given donor appeared exclusively in a single fold.

We trained and evaluated multiple model architectures: logistic regression, random forest, ElasticNet, Explainable Boosting Machines (EBMs) [12], and XGBoost [13]. For each model architecture, hyperparameters were tuned via the hyperopt [14] package using 10‐fold cross‐validation. Within each cross‐validation resample, 80% of the training folds were used for model training, 20% were used for hyperopt tuning, and the hold‐out fold was used for evaluation.

Given the large class imbalance, we opted to grade prediction performance based on average precision instead of area under the receiver operating characteristics curve (AUROC). In our evaluations, we found that the AUROCs were deceptively high (AUROCs > 0.80) despite relatively poor precision and recall characteristics. Instead, we concluded that the precision–recall curves (PRCs) were more informative for estimating the clinical utility of the models. Given our goal of using models to assist with allocation decisions or expedited placements, high precision is important to avoid offering the organ to programs that would ultimately decline. Therefore, we chose average precision for evaluation, as it summarizes PRCs into a single metric.

Based on the cross‐validation results, we selected the best‐performing model for additional experimentation. We produced 4 sets of additional features that were each separately included in the model training and evaluation alongside the standard features.

1. Historical Organ Procurement Organization (OPO) characteristics (e.g., number of donors allocated in the last year)
2. Historical transplant hospital (TXH) characteristics (e.g., number of transplants performed in the last year)
3. Historical characteristics about the OPO and TXH pair (e.g., number of acceptances by this program for this OPO in this last year)
4. Transportation‐related information about the OPO (e.g., number of commercial airports in a 50‐mile radius)

To address class imbalance, we also applied a sampling procedure to produce a balanced training dataset. We first performed random undersampling to produce a 10:1 class imbalance, followed by oversampling via ProWRAS [15] to produce the 1:1 fully balanced dataset. The sampling strategy was chosen based on a sensitivity analysis on the training data as reported in Table 1. Model performances for all experiments were evaluated on the unseen test set.

**Table 1:** Sensitivity test on training data for different sampling strategies.

Type of sampling	Undersample ratio	Oversample ratio	AUROC	Average precision
Undersampling	10:1	—	0.884	0.139
	1:1	—	0.882	0.117

Oversampling	—	10:1	0.880	0.146
	—	1:1	0.877	0.143

Undersampling then oversampling	50:1	10:1	0.880	0.145
	50:1	1:1	0.878	0.144
	10:1	1:1	**0.889**	**0.148**

No sampling	—	—	0.879	0.145

*Note:* AUROC: Area under the receiver operating characteristics curve. The best scores across all sampling strategies are presented in bold.

All analyses were conducted via Python 3.11 using the Databricks 14.3 Machine Learning Runtime. All models were implemented using the Apache Spark (3.5.0) MLLib implementations of the distributed models except for the EBMs (interpretml 0.28.0) [12] and XGboost (xgboost 1.7.5) [13] models, which were provided by their respective Python packages. Hyperopt [14] was included in the Databricks runtime at Version 0.2.7.

### 2.3. Baseline Features

Our starting baseline features for all models were largely chosen from the offered models used for risk‐adjusted program evaluation [7]. Categorical features were one‐hot encoded, and numerical features were standardized and scaled. To handle missing variables, we added a “Missing” category for categorical features and a median imputation for numeric variables. The one‐hot encoding, standardization, scaling, and imputation were all performed either on the training folds during 10‐fold cross validation or on the entire training set for final model evaluation; these transformations were then applied to the final hold‐out fold or final test set.

As many of the model architectures tested incorporated regularization (such as the logistic regression models via L1 and/or L2 penalties) or were robust to multicollinearity (e.g., random forests, which randomly select subsets of features), we opted not to perform feature selection such that we could assess feature importance across all input features.

### 2.4. Explainer

To understand how the models make predictions, we applied a model interpretation approach called SHAP [16] to the best‐performing model. SHAP generates values representing the relative contribution of each feature on the output of the trained classifier to test examples. In addition, we also used a generalized additive model (GAM)‐based classifier named EBM, which provides interpretable relative weights of the features used in the trained model [12]. While this provides an arguably more reliable interpretation of the corresponding classifier as opposed to the inherently approximate nature of SHAP, it requires training a standalone classifier itself. We retrained the best‐performing model using only the top 15 features identified by SHAP. We also extracted the top 15 features identified by the ElasticNet model regularization path for comparison to the variables identified by the explainers.

### 2.5. Equity Analysis

We analyze the best‐performing model through an equity lens by comparing various measures of predictive performance across subgroups of individuals: precision, recall, and false positive rate. The socio‐demographic characteristics of individuals that we considered were race, gender, age, and financial status. For each subgroup, we also evaluate the offer acceptance prior from the data to understand whether certain subgroups tend to accept offers more proactively than others. The offer acceptance prior probability is computed as the percentage of offers seen by individuals from a subgroup that are accepted.

## 3. Results

### 3.1. Model Performance

**Figure 3:**
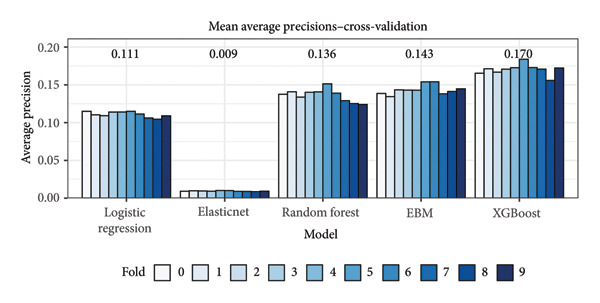
Cross‐validation results from training various model architectures. Based on the mean average precisions noted above for each model, the XGBoost model was selected as the best model for additional experimentation. EBM means Explainable Boosting Machine.

**Figure 4:**
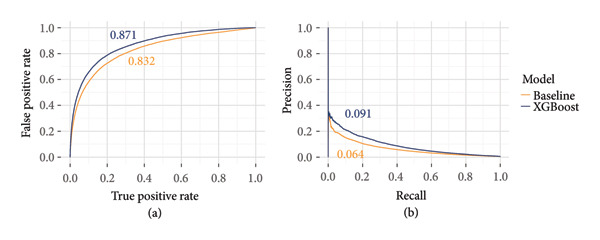
Receiver‐operator characteristics (ROC) curves (a) and precision–recall (PR) curves (b) for the baseline model and the best‐performing model (XGBoost) as evaluated on the unseen test set. Area under ROCs (AUROC) and average precisions are noted next to each curve.

Based on the mean average precision from 10‐fold cross‐validation on the training set, the XGBoost model outperformed all other models (Figure 3) and was selected as the best model for further experimentation. Receiver operating characteristic (ROC) curves and precision–recall (PR) curves based on the test set are shown for the baseline and XGBoost models in Figure 4.

**Figure 5:**
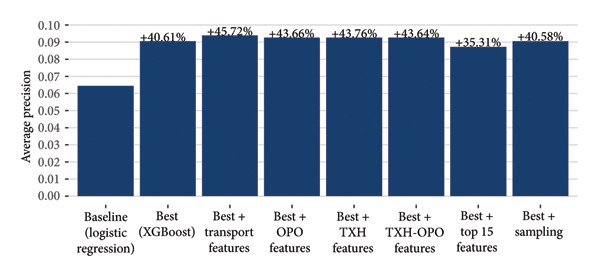
Average precision metric calculated on the test set for various models. The baseline model is a logistic regression model, and the best model was selected for having the highest cross‐validation metrics calculated on the training set (Figure 3). We experimented with both sampling and additional feature engineering for the best model and evaluated performance on the same test set.

The XGBoost model performed 40.6% better than a simple logistic regression model, and each set of additional features improved performance on test set predictions, albeit not substantially (Figure 5). Including transportation features increased performance of the best model by approximately 45.7% over baseline, the largest improvement observed. Including other institutional features related to the OPO, TXH, or OPO‐TXH pair, all produced similar performance gains of around 43.7%. Despite the relative improvements, absolute performance across all experiments was low, with a maximum average precision of 0.0940.

Without sampling, the class imbalance for our dataset was approximately 105:1 (105 not‐accepted offers for every accepted offer). A combination of undersampling followed by oversampling produced a 1:1 balanced training dataset. Figure 5 shows the performance of a model trained on the balanced dataset and evaluated on the unbalanced holdout data. Our sampling procedure did not noticeably improve model performance (average precision of 0.0907 both with and without sampling).

### 3.2. Explainer

**Figure 7:**
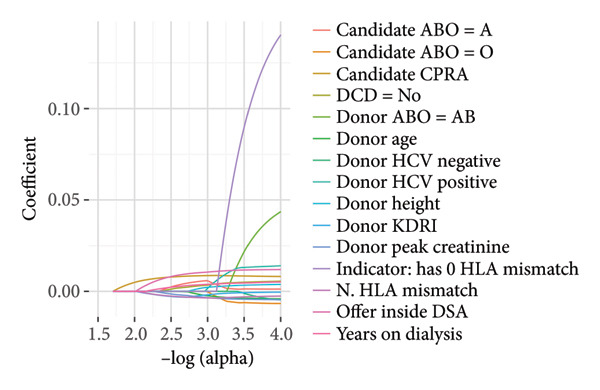
Regularization path for the ElasticNet model used. Only the top 15 features with the largest coefficient absolute values are displayed (CPRA: calculated panel reactive antibody; DCD: donation after circulatory death; HCV: hepatitis C; KDRI: kidney donor risk index; HLA: human leukocyte antigen; DSA: donor service area).

We obtained the list of the top 15 contributing features from EBM and the SHAP explainer. These features, along with their relative weights, are shown in Figure 6. Many of the top 15 features from both approaches overlap with features used in the SRTR risk‐adjustment models for offer acceptance, including years on dialysis, donor age, and CPRA, among others. Pumping parameters such as pump flow and resistance were identified as impactful features; however, these factors are not currently used in the risk‐adjustment models. While some variables have a clear association with model impact (for example, low donor age being correlated with positive model predictions, i.e., accepted offers), others have mixed effects, such as kidney glomerulosclerosis. For comparison, the top 15 features from the ElasticNet model regularization path are shown in Figure 7, and we observed overlap among some of the clinical and demographic variables, such as creatinine and donor age. Training a model on only the top 15 features identified by SHAP resulted in better performance than the baseline but worse than including all variables (Figure 5).

Figure 6a–b. Feature impacts of the top 15 features (in descending order of impact) according to the SHapley Additive exPlanations (SHAP) explainer and Explainable Boosting Machines (EBM) model. (DSA: donor service area, LKI: left kidney, RKI: right kidney, CPRA: calculated panel reactive antibody; EPTS: expected post‐transplant survival; KDRI: kidney donor risk index; HLA: human leukocyte antigen; see Table S1 for feature details.) (a) For each feature, the SHAP values (*x*‐axis) represent the impact of individual data samples (represented by each point) with different feature values (colormap). A higher SHAP value is associated with a higher probability of offer acceptance. For example, low peak serum creatinine was more associated with offer acceptance (positive model impact). (b) For EBM, the mean absolute values of the weights associated with different features are shown (^∗^denotes an interaction).

**Figure:**
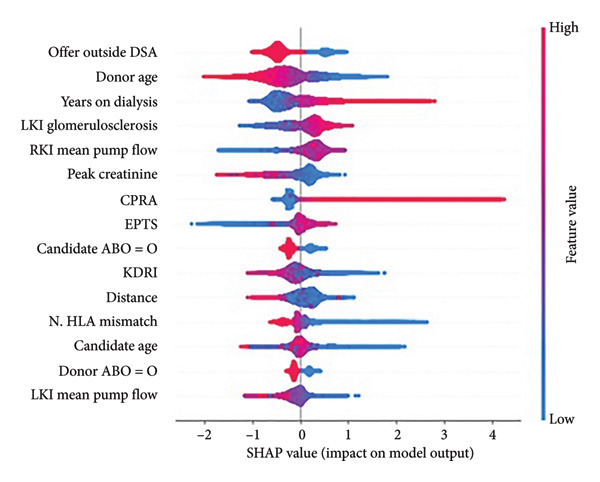
(a)

**Figure:**
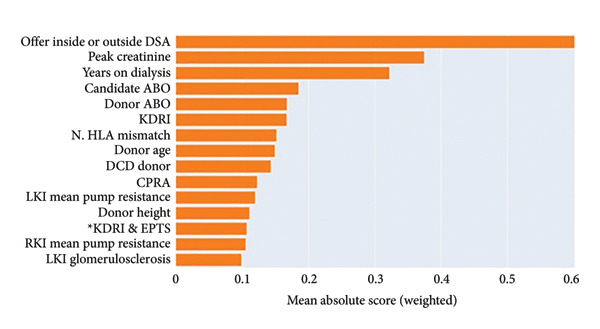
(b)

### 3.3. Equity Analysis

Results of stratified model performance across various candidate demographics are shown in Table 2. Performance was similar for public and private insurance. Offers to female candidates had a higher false positive rate than male candidates (0.00040 vs. 0.00017) despite offers to female candidates being accepted at higher rates (1.20% vs. 0.85%). In terms of precision, recall, and false positive rates, the model performed worse for offers to Black candidates than any other race/ethnicity grouping (besides the small “Other” category) despite similar offer volume and acceptance rates in Black and White candidates. Across age demographics, the model performed poorly for pediatric candidates, with no accepted offers being correctly predicted; however, pediatric candidates also had low total offer volume at less than 1% of all offers. Across adult age categories, model performance was largely uniform.

**Table 2:** Model performance metrics stratified by equity‐relevant socio‐demographic factors.

	Count	% of offers	Precision	Recall	False positive rate (%)	Offer acceptance prior (%)
Gender						
Female	668,275	32.36	0.349	0.018	0.040	1.20
Male	1,396,442	67.63	0.341	0.010	0.017	0.85
Race and ethnicity						
Asian	174,881	8.47	0.351	0.009	0.014	0.84
Black	692,332	33.53	0.255	0.010	0.029	0.99
Hispanic	477,020	23.10	0.401	0.014	0.017	0.83
Non‐Hispanic multiracial	12,471	0.60	0.400	0.012	0.024	1.40
White	685,697	33.21	0.398	0.018	0.018	1.00
Other	22,316	1.08	0.200	0.003	0.028	1.40
Age range						
0–1	88	0.004	0	0	0	0.00
1–5	2302	0.11	0	0	0	0.96
6–10	2249	0.11	0	0	0.045	1.20
11–17	4655	0.22	0	0	0.022	1.10
18–34	144,319	6.99	0.321	0.012	0.025	0.98
35–49	449,665	21.78	0.355	0.015	0.026	0.96
50–64	921,590	44.63	0.351	0.013	0.023	0.96
65+	539,849	26.15	0.338	0.014	0.026	0.97
Primary payment						
Public	1,150,634	55.73	0.345	0.014	0.025	0.97
Private	910,469	44.10	0.346	0.013	0.024	0.96
Other	3604	0.17	0	0	0	1.10

## 4. Discussion

### 4.1. Overall Performance

On our test set, despite deceptively high AUROCs (Figure 4), our models never eclipsed a 10% average precision (Figure 5). At the 10% recall threshold, the baseline and XGBoost models achieved precisions of 15% and 21%, respectively (Figure 4). As we describe below, we do not believe this meets a threshold for clinical application, but we should consider what the application is and which takeaways are relevant for iterating toward practical usage. As kidney nonuse rates continue to rise, one potential application of offer acceptance models is to prioritize allocation toward specific candidates that will accept a given kidney, that is, reorder the match.

### 4.2. Clinical Relevance to Allocation

While we want to increase model performance, it is not clear which precision/recall thresholds would qualify as clinically relevant. A device to diagnose diabetic retinopathy is regarded as the first AI‐powered device with Food and Drug Administration (FDA) approval to be operated without corroborating review by a physician. The qualifying studies graded its predictions against human clinicians, where it performed similarly or better in selected metrics [17]. Similarly, we could require that the offer acceptance model perform at least as well as whatever system it is replacing or augmenting. In the match reordering context, we would expect the model to place organs “better” than the current allocation policy (KAS250) [18].

For accepted kidneys, the mean, median, and 75^th^ percentile of the sequence number of the accepted offer are 665, 18, and 141, respectively, under KAS250 [18]. So, although most kidneys are placed relatively early in the match, some kidneys need to be offered to hundreds or thousands of candidates. Kidneys requiring many offers may accrue additional cold ischemic time, which could negatively impact graft function or discourage transplantation. A limitation to this study is that we did not rigorously assess how many offers these models would take to find a match. As a crude estimate, assume a 20% precision translates to 20/100 = 5 offers to place an organ. That may be a potential improvement in allocation priority for kidneys that would otherwise require hundreds of offers. However, a more comprehensive study is needed to assess if reordering offers to candidates with a high probability of acceptance would accept the organ in fewer offers than in the traditional policy‐driven ordering. As is, the model is not performant at identifying a specific candidate to accept an offer, especially when taking into account the equity considerations that are baked into allocation policy.

### 4.3. Allocation Equity

Allocation policies are developed with more intention than just placing an organ quickly; there are various clinical and demographic equity considerations, such as giving priority to pediatric candidates or highly sensitized candidates. Given the low recall of both models, it is likely that these equity considerations would not be respected by the models out‐of‐the‐box. That is, the model may place an organ within 5 offers but skip candidates who are currently at the top of the match due to clinical and equity prioritization. Predictive models used in allocation should be judged not just in terms of placing organs quickly but also in terms of the community‐driven allocation priorities.

One of the priorities of the Kidney Allocation System (KAS; effective December 4, 2014 [19]) and the current Kidney Allocation System with 250 nautical mile acuity circles (KAS250; effective March 15, 2021 [18]) has been to improve access to kidney transplants amongst underserved populations [18, 20–22]. While this prioritization has resulted in an increased number of Black and Hispanic individuals receiving deceased donor transplants [19, 20], disparities continue to exist throughout the transplant pathway [18, 19, 23–25], highlighting the need for further efforts toward equitable access to transplant care. Such scrutiny of equity is also imperative for algorithmic or statistical modeling approaches that aim to simplify or improve kidney allocation. Some recent statistical models developed for prediction tasks have included race as an input feature to the model [23, 26] but have failed to consider the impact of the model’s predictions on individuals from ethnic/racial minorities [26].

Our own analysis of model performance on the equity‐relevant socio‐demographic features revealed that the model would underperform with some candidate demographics. There were no correctly predicted accepted offers for pediatric candidates, and performance for Black candidates was noticeably lower than for White candidates despite having similar offer volumes and acceptance rates. Specialized models for pediatric candidates may be appropriate given the low offer volume (less than 1% of our dataset), and further tuning may be necessary to ensure equitable performance across other demographic groups. Otherwise, these models risk deprioritizing both underserved populations and medically complex patients that would otherwise rise to the top of the match.

### 4.4. Other Challenges

Evaluating an organ offer for transplant is a complex, multifaceted process that is difficult to model, and we identified challenges and areas for improvement. Class imbalance is prominent in this context, especially for medically complicated kidneys, where dozens to hundreds of transplant programs may reject the organ for all their candidates. The under‐ and oversampling procedure we implemented did not noticeably improve results, but further tuning of the sampling procedure (different class ratios, different algorithms, etc.) may still provide benefit. Further, the decision to accept and transplant an organ involves multiple personnel, including coordinators, medical specialists, and surgeons, and the OPTN data do not record who was involved in the decision process, which may be relevant for the prediction model.

Changes in allocation policy and transplant technology also present challenges for training predictive models. We chose the start of our cohort to be after KAS implementation, which was a significant shift in kidney allocation. Most of the cohort was before the emergence of COVID‐19, which also impacted allocation. However, our training set only includes approximately 1 year of COVID‐era and KAS250‐era allocations, whereas our test set is entirely post‐KAS250 implementation and after COVID‐19 was declared an emergency. Besides those explicit policy and public health changes, we also know that gradually the donor pool is expanding and includes many harder‐to‐place donors [27]. These temporal factors may adversely impact our evaluation metrics on the test set.

Neural networks (NNs), such as deep NNs, have been developed for other clinical and healthcare applications, and their use for offer acceptance prediction should be evaluated. We chose not to evaluate them as part of this work, as developing NNs would benefit from different optimization strategies and specialized computing hardware. There are more potential NN architectures than models we tested, and each NN architecture would require hyperparameter tuning in addition to training. Although NNs often achieve state‐of‐the‐art performance with unstructured data, it is not clear if there is any performance gain from applying NNs on highly heterogeneous tabular data (such as our offer dataset) as compared to machine learning models like boosted decision trees [28]. A dedicated study should be done to maximize the utility of an NN model.

Instead, we focused on evaluating multiple linear and tree‐based models. Some of these models make assumptions about the underlying dataset, such as each observation (each offer) being statistically independent from other observations. A limitation for this study is that we opted solely to assess overall predictive performance. Future evaluation of these assumptions may help researchers decide which models are appropriate for their context in addition to performance metrics.

### 4.5. Recommendations

Despite the challenges, we have recommendations for developing an offer acceptance predictive model based on our improved performance over the baseline model. Factors beyond the standard demographic and clinical variables proved useful for predictions. We observed the largest performance increase by including transportation features such as airport proximity; this is especially relevant since 2021, as KAS250 resulted in kidneys being shared in wider geographic areas [11, 18].

Institutional history, such as the number of transplants a program performed in the previous year, also helped to predict current offer acceptance decisions. These performance gains suggest that operational factors not captured in standard transplant datasets are relevant for predictive models.

We did not see the anticipated gains from undersampling followed by oversampling, so class imbalance remained a challenging problem that warrants further investigation. Performance based on AUROCs is deceptive in this setting, so we considered precision and recall metrics instead. Rather than sampling, it may be advantageous to reframe the modeling problem such that instead of predicting which specific candidate accepts an offer, a model is used to predict more generally if the kidneys will be accepted by a certain sequence number (i.e., accepted early or late in the match). This would prevent such an imbalanced dataset while still providing potentially actionable predictions that could inform whether an OPO should trigger an expedited allocation pathway.

Regardless of the modeling approach, we believe that any evaluation should incorporate equity analysis to ensure that models align with community‐driven allocation priorities. For example, we noted that our models did not correctly identify any accepted offers to pediatric candidates, which, along with low pediatric offer volume, indicates we should perhaps model those offers separately.

These performance and equity factors should be considered for any predictive model being used for transplant decision support or for expedited placement workflows.

**Nomenclature**
OPTN: Organ Procurement and Transplantation Network
DCD: Donation after circulatory death
CPRA: Calculated panel reactive antibody
SRTR: Scientific Registry of Transplant Recipients
EBM: Explainable Boosting Machines
EPTS: Expected posttransplant survival
KDRI: Kidney donor risk index
OPO: Organ Procurement Organizations
TXH: Transplant hospital
ROC: Receiver operating characteristic
AUROC: Area under the receiver operating characteristics curve
PRC: Precision–recall curve
GAM: Generalized additive model
FDA: Food and Drug Administration
KAS: Kidney Allocation System
KAS250: Kidney Allocation System with 250‐nautical‐mile acuity circles
NN: Neural network

## Conflicts of Interest

The authors declare no conflicts of interest.

## Author Contributions

Carlos Martinez, Juan Lavista Ferres, Rahul Dodhia, and William B. Weeks participated in the research design.

Md Nasir, Meghana Kshirsagar, Cass McCharen, Rae Shean, and William B. Weeks participated in reviewing the writing and analyses.

Carlos Martinez, Md Nasir, Meghana Kshirsagar, and Cass McCharen participated in the data analysis.

Carlos Martinez, Md Nasir, and Meghana Kshirsagar contributed to drafting the manuscript.

## Funding

No funding was received for this manuscript.

## Supporting Information

Supporting Table S1: List of features.

## Supporting information


**Supporting Information** Additional supporting information can be found online in the Supporting Information section.

## Data Availability

The data that support the findings of this study are available from the Organ Procurement and Transplantation Network. Restrictions apply to the availability of these data, which were used under license for this study. Data are available from https://optn.transplant.hrsa.gov/data/view-data-reports/request-data/ with the permission of the Organ Procurement and Transplantation Network.
