# Supplementary material for: Predictive Models for Kidney Offer Acceptance: Challenges and Strategies
Source: J Transplant. 2026 Jan 9;2026:8243450. doi: 10.1155/joot/8243450 (PMC12784377; doi:10.1155/joot/8243450)
Supplement: Supplementary file 1 — Supporting Information Additional supporting information can be found online in the Supporting Information section. [file JOOT-2026-8243450-s001.docx]

SDC, Table S1: List of features.

| Which model(s) | Feature name | Description |
| --- | --- | --- |
| All models | Dialysis | Number of years the candidate has been on dialysis |
|  | KDRI | Kidney Donor Risk Index; median method |
|  | EPTS | Estimated post-transplant survival score |
|  | D. Age | Donor age years |
|  | C. Age | Candidate age in years |
|  | BSA Ratio | Ratio of candidate BSA to donor BSA |
|  | Distance | Distance between donor hospital and transplant hospital (kilometers) |
|  | N. HLA Mismatch | Number of HLA mismatches between the donor and candidate |
|  | CPRA | Candidate CPRA |
|  | D. Height | Donor height (ft) |
|  | D. Weight | Donor weight (lbs) |
|  | C. Height | Candidate height (cm) |
|  | C. Weight | Candidate weight (kg) |
|  | Creatinine - High | Peak creatinine at the time of offer |
|  | LKI Pump Flow - Mean | Mean pump flow for the left kidney |
|  | RKI Pump Flow - Mean | Mean pump flow for the right kidney |
|  | LKI Pump Resistance - Mean | Mean pump resistance for the left kidney |
|  | RKI Pump Resistance - Mean | Mean pump resistance for the right kidney |
|  | LKI GS | Percent of glomeruli that are sclerosed (glomerulosclerosis) in left kidney |
|  | RKI GS | Percent of glomeruli that are sclerosed (glomerulosclerosis) in right kidney |
|  | Double KI | Indicator for en bloc or dual offer vs single-kidney offer |
|  | 0 ABDR | If there were 0 mismatches between the donor and candidate |
|  | KP Candidate | If the candidate is also a Kidney-Pancreas candidate |
|  | Local | If the donor and candidate are located in the same DSA (served by the same OPO) |
|  | Nighttime | Offer decision happened between 6 PM and 6 AM |
|  | Weekend | Offer decision happened on a Saturday or Sunday |
|  | Risk | Donor has risk factors for blood-borne disease transmission |
|  | D. ABO | Donor ABO |
|  | C. ABO | Candidate ABO |
|  | Cardiac Duration | Duration of donor's cardiac arrest |
|  | HCV | Donor HCV Status |
|  | C. Diagnosis | Candidate's primary diagnosis for transplant |
|  | D. COD | Donor cause of death |
|  | D. circumstance | Donor death circumstance |
|  | DCD | DCD Indicator |
|  | Cardiac Arrest | If the donor had a cardiac arrest |
|  | Hypertension | The donor's history of hypertension |
|  | Diabetes | The donor's history of diabetes |
|  | Cancer | The donor's history of cancer |
|  | IV Drugs | The donor's history of I.V. drug usage |
| TRANSPLANT hospital & Organ PROCUREMENT organization (TXH-OPO) | Duration: xclamp to accept | Average time between final acceptance and cross clamp |
|  | Duration: dialysis | Average years the candidates are on dialysis |
|  | CPRA - Mean | Average CPRA |
|  | Distance - Mean | Average distance between hospitals |
|  | Sequence Number - Mean | Average sequence number of candidates on the list |
|  | N. Donors | N. donors with offers sent |
|  | N. Candidates | N. candidates that received offers |
|  | N. Matches | N. matches from this OPO where this TXH received offers |
|  | N. Offers | N. offers from this OPO to this TXH |
|  | EPTS - Mean | Average EPTS |
|  | KDPI - Mean | Average KDPI |
|  | N. Transplants | N. transplants at this TXH from donors at this OPO |
|  | N. Accepted | N. accepted offers |
|  | N. Declined | N. declined offers |
|  | N. Bypassed | N. bypassed offers |
|  | N. PY | N. provisionally accepted offers |
|  | N. PY and Accept | N. provisionally accepted then accepted offers |
|  | Ratio: donors to candidates | Number of donors divided by number of candidates offered |
|  | Ratio: offers to candidates | Number of offers divided by the number of candidates offered |
|  | Ratio: transplants to candidates | Number of transplants divided by patients offered |
|  | Ratio: transplants to matches | Number of transplants divided by number of matches |
|  | Ratio: candidates to matches | Ratio of the number of patients divided by the number of matches |
|  | Percent transplant | Percent of offers that led to transplant |
|  | Percent declined | Percent of offers that had a final response of No |
|  | Percent bypass | Percent of offers that had a final response of Bypass |
|  | Percent PY | Percent of offers that had an initial response of Provisional Accept |
|  | Percent PY and accepted | Percent of offers that were both provisionally and ultimately accepted |
|  | OPO Ratio: percent PY and accepted | Percent of offers that were both provisionally and ultimately accepted between this OPO and TXH divided by the percent of all offers to this OPO that were provisionally then ultimately accepted |
|  | OPO Ratio: percent declined | Percent of offers that had a final response of No divided by the percent of all offers from this OPO that were declined |
|  | OPO Ratio: percent bypass | Percent of offers that had a final response of Bypass divided by the percent of all offers from this OPO that were bypassed |
|  | OPO Ratio: percent transplant | Percent of offers that led to transplant divided by the percent of all offers from this OPO that led to transplant |
|  | OPO Ratio: donors/candidates | Ratio of (the number of donors divided by number of candidates offered) to the total number of donors at this OPO |
|  | OPO Ratio: candidates | Ratio of the number of candidates at this TXH to the number of candidates offered in total by the OPO |
|  | OPO Ratio: donors | Ratio of the number of donors with offers received at this TXH to the number of donors recovered by the OPO |
| Transplant Hospital (TXH) | Region | OPTN Region of the transplant hospital |
|  | Duration: clamp to response | Average time between transplant hospital final response and cross clamp |
|  | Duration: dialysis | Average years on dialysis |
|  | CPRA - Mean | Average CPRA |
|  | Distance - Mean | Average distance to the donor hospital |
|  | Sequence Number - Mean | Average sequence number of candidates on the list |
|  | N. Donors | Number of donors from which the TXH received offers |
|  | N. Candidates | Number of candidates |
|  | N. Matches | Number of matches on which the TXH was offered |
|  | N. Offers | Number of offers received |
|  | EPTS - Mean | Average EPTS of offers |
|  | KDPI - Mean | Average KDPI of offers |
|  | N. Transplants | Number of transplants performed |
|  | N. Accepted | Number of offers accepted |
|  | N. Declined | Number of offers declined |
|  | N. Bypassed | Number of offers bypassed |
|  | N. PY | Number of offers provisionally accepted |
|  | N. PY and accept | Number of offers provisionally accepted and then accepted |
|  | Ratio: donors to candidates | Ratio of number of donors from which offers were received to the number of candidates that received offers |
|  | Ratio: offers to candidates | Ratio of the number of offers received to the number of candidates |
|  | Ratio: transplants to candidates | Ratio of the number of transplants performed to the number of candidates that received offers |
|  | Ratio: transplants to matches | Ratio of the number of transplants to the number of matches where the TXH received offers |
|  | Ratio: offers to matches | Ratio of the number of candidates that received offers to the number of matches where the TXH received offers |
|  | Ratio: accepts to matches | Ratio of the number of acceptances to the number of matches where offers were received |
|  | Percent transplant | Percent of offers that led to transplant |
|  | Percent declined | Percent of offers that were declined |
|  | Percent bypassed | Percent of offers that were bypassed |
|  | Percent PY | Percent of offers that were provisionally accepted |
|  | Percent PY and accept | Number of offers provisionally accepted and then accepted |
| Organ procurement Organization (OPO) | Duration: cross clamp to accept | Average time from cross clamp to accept |
|  | Duration: dialysis | Average years on dialysis |
|  | CPRA - Mean | Average CPRA |
|  | Distance - Mean | Average distance between hospitals |
|  | Sequence number - Mean | Average sequence number of candidates on the match run |
|  | N. Donors | Number of donors for which matches were initiated |
|  | N. Candidates | Number of candidates that were sent offers |
|  | N. Matches | Number of matches initiated |
|  | N. Offers | Number of offers sent |
|  | EPTS - Mean | Average EPTS |
|  | KDPI - Mean | Average KDPI |
|  | N. Transplant | Number of transplants |
|  | N. Accepted | Number of accepted offers |
|  | N. Declined | Number of declined offers |
|  | N. Bypassed | Number of bypassed offers |
|  | N. PY | Number of provisionally accepted offers |
|  | N. PY and Accept | Number of provisionally accepted offers that were ultimately accepted |
|  | Ratio: donors to candidates | Ratio of the number of donors to number of candidates offered |
|  | Ratio: offers to candidates | Ratio of the number of offers to the number of candidates offered |
|  | Ratio: transplants to candidates | Ratio of the number of transplants to the number of patients offered |
|  | Ratio: transplants to matches | Ratio of number of transplants to the number of matches initiated |
|  | Ratio: candidates to matches | Ratio of the number of candidates sent offers to the number of matches initiated |
|  | Ratio: offers to matches | Ratio of the number of accepted offers to the number of matches initiated |
|  | Percent transplanted | Percent of offers sent that resulted in transplant |
|  | Percent declined | Percent of the number of offers sent that resulted in decline |
|  | Percent bypassed | Percent of the number of offers sent that were bypassed |
|  | Percent PY | Percent of the number of offers sent that were provisionally accepted |
|  | Percent PY and accept | Percent of the number of offers sent that were provisionally accepted and ultimately accepted |
| Transportation | Local non-use | Percent of kidneys not used in 250NM radius around the TXH |
|  | Population density | Population density around the TXH |
|  | Airport distance | Kilometers to closest airport |
|  | Flight density | Average number of daily flights around the TXH |
|  | Seat density | Average number of commercially available seats per flight at the closest airport to the TXH |
